# Using a Quality-Controlled Dataset From ViSi Mobile Monitoring for Analyzing Posture Patterns of Hospitalized Patients: Retrospective Observational Study

**DOI:** 10.2196/54735

**Published:** 2024-11-06

**Authors:** Emily J Huang, Yuexin Chen, Clancy J Clark

**Affiliations:** 1 Department of Statistical Sciences, Wake Forest University, 1834 Wake Forest Road, Winston Salem, NC, 27109, United States, 1 336 758 5300; 2 Department of Surgery, Wake Forest School of Medicine, Winston Salem, NC, United States

**Keywords:** posture monitoring, ViSi mobile, wearable device, inpatient, quality control, observational study, monitoring data, inpatient monitoring, wearables, posture

## Abstract

**Background:**

ViSi Mobile has the capability of monitoring a patient’s posture continuously during hospitalization. Analysis of ViSi telemetry data enables researchers and health care providers to quantify an individual patient’s movement and investigate collective patterns of many patients. However, erroneous values can exist in routinely collected ViSi telemetry data. Data must be scrutinized to remove erroneous records before statistical analysis.

**Objective:**

The objectives of this study were to (1) develop a data cleaning procedure for a 1-year inpatient ViSi posture dataset, (2) consolidate posture codes into categories, (3) derive concise summary statistics from the continuous monitoring data, and (4) study types of patient posture habits using summary statistics of posture duration and transition frequency.

**Methods:**

This study examined the 2019 inpatient ViSi posture records from Atrium Health Wake Forest Baptist Medical Center. First, 2 types of errors, record overlap and time inconsistency, were identified. An automated procedure was designed to search all records for these errors. A data cleaning procedure removed erroneous records. Second, data preprocessing was conducted. Each patient’s categorical time series was simplified by consolidating the 185 ViSi codes into 5 categories (Lying, Reclined, Upright, Unknown, User-defined). A majority vote process was applied to remove bursts of short duration. Third, statistical analysis was conducted. For each patient, summary statistics were generated to measure average time duration of each posture and rate of posture transitions during the whole day and separately during daytime and nighttime. A k-means clustering analysis was performed to divide the patients into subgroups objectively.

**Results:**

The analysis used a sample of 690 patients, with a median of 3 days of extensive ViSi monitoring per patient. The median of posture durations was 10.2 hours/day for Lying, 8.0 hours/day for Reclined, and 2.5 hours/day for Upright. Lying had similar percentages of patients in low and high durations. Reclined showed a decrease in patients for higher durations. Upright had its peak at 0‐2 hours, with a decrease for higher durations. Scatter plots showed that patients could be divided into several subgroups with different posture habits. This was reinforced by the k-means analysis, which identified an active subgroup and two sedentary ones with different resting styles.

**Conclusions:**

Using a 1-year ViSi dataset from routine inpatient monitoring, we derived summary statistics of posture duration and posture transitions for each patient and analyzed the summary statistics to identify patterns in the patient population. This analysis revealed several types of patient posture habits. Before analysis, we also developed methodology to clean and preprocess routinely collected inpatient ViSi monitoring data, which is a major contribution of this study. The procedure developed for data cleaning and preprocessing can have broad application to other monitoring systems used in hospitals.

## Introduction

Monitoring the level of mobilization and its changes over time provides critical health information on hospitalized patients. For example, timing of resuming certain postures after surgery can reflect the pace and quality of recovery [1,2]. A traditional method for tracking posture is direct observation, which can be done by a nurse who directly observes and records the patient’s posture manually. However, this is time-consuming and resource-intensive, especially when the observers are tracking multiple patients on a ward [3]. Other limitations of direct observation are subjective reports and intermittent recordings, which may also suffer from bias and random human error [1,4].

To overcome these limitations, some hospitals utilize monitoring systems that use sensors (eg, accelerometer, infrared, and radio frequency identification sensors) to collect measurements for tracking a patient’s posture or movement [1,3,5-7]. The collection of measurements by these sensors is continuous and automated. A trained algorithm in the device converts the collected sensor data to a patient’s posture objectively. The output reports the patient’s postures and activities instantaneously. These data can be used to derive the amount of time a patient spends on bed rest. Moreover, posture monitoring records before and after surgery could be compared to identify whether a patient is having a smooth recovery or a poor one that requires intervention.

Statistical analyses of large datasets collected from many patients using these monitoring systems can further reveal patterns in patients’ condition and behavior that can be useful for medical research and operational guidance. There have been preliminary results in connecting hospital sensor observations and patients’ condition statistically. For example, recent accelerometer-based studies found that inpatients with acute illness were highly inactive, spending 93%-98.8% of their stay sedentary [5]. Researchers have also used posture monitoring to capture changes in a patient’s mobility over the course of their hospital stay and use it as an indicator of recovery rate. In a study of inpatients aged 60 years or older admitted from the emergency room, Theou et al found that those patients classified as less mobile upon admission experienced an increase in their upright time during their hospital stay, possibly corresponding to an improvement in their health condition [8].

A real-time monitoring system that has demonstrated promise in the hospital setting is ViSi Mobile (Sotera Wireless) [9-12]. This device continuously monitors a patient’s vital signs and posture. It can be used to capture walking periods, as well as more subtle movements, such as getting out of bed or changing posture in bed. To the best of our knowledge, there are only 3 published studies analyzing ViSi posture data of hospitalized patients. Restrepo et al tested the accuracy of ViSi posture measurements by comparing them to those from direct observation [3]. This study found that ViSi can accurately classify certain static postures, such as lying down and sitting. However, it can have systematic errors in classifying the activity of walking. In an analysis of 2 randomized trials with patients recovering from abdominal surgery, Rivas et al examined the relationship between self-reported pain score and ViSi-measured mobility [1]. The authors estimated the decreasing rate of change in mobility with increasing pain score, and they also found that lower mobility was associated with more postoperative complications. In a study of ViSi data from noncardiac surgery patients, Turan et al found that increased mobility in the 48 hours postoperation was associated with fewer postoperative complications and shorter length of hospital stay [13].

This study sought to characterize posture habits in a large hospitalized patient population, including both surgery and nonsurgery patients. Another purpose of the study was to conduct quality control of the ViSi posture data because the routine hospital measurements originally collected for operational purposes contain different types of errors that require careful treatment. Overall, the study objectives were to (1) develop a data cleaning procedure for a large inpatient ViSi posture monitoring dataset with a duration of 1 year, (2) consolidate posture codes into categories, (3) derive concise summary statistics from the continuous monitoring data, and (4) study types of patient posture habits using summary statistics of posture duration and transition frequency.

## Methods

### Ethical Considerations

This study used a deidentified dataset and was approved by the Wake Forest School of Medicine Institutional Review Board (IRB00051033). No patient consent was required. No compensation was provided.

### Data Description

Since 2015, real-time posture data have been collected routinely using the ViSi Mobile System for patients on postoperative surgical wards and acute medical inpatient wards at Atrium Health Wake Forest Baptist Medical Center in North Carolina, United States of America. The starting point of ViSi monitoring is at admission to the hospital ward. The ViSi device has a wrist module, upper arm module, and chest module, each of which contains a 3-axis accelerometer [14]. Posture is estimated using data collected by the accelerometer sensors. In addition, other sensors on the ViSi device simultaneously measure blood pressure, heart rate, pulse rate, respiratory rate, and oxygen saturation.

This study focused on the ViSi posture data collected at Atrium Health Wake Forest Baptist Medical Center in 2019 from both surgery and medicine services. The ViSi Mobile system converts accelerometer measurements into 15 categories of posture using a proprietary algorithm developed by Sotera Wireless. Each category is presented by a code (Multimedia Appendix 1). A code can describe a single activity, such as walking (WLK). Some codes may combine more than one position or situation into one posture category. For instance, code U90 for upright can be sitting or standing. Code U45, representing a reclined position, may correspond to sitting in a reclined chair or lying when the bed is in a reclined position. On the other hand, for lying down, multiple codes are used to distinguish different postures, such as supine (LSP), prone (LPR), right side (LRS), and left side (LLS). FALL indicates that a patient may have fallen (this code was not observed in the 2019 ViSi posture dataset). UNK indicates that the patient’s posture could not be determined by the proprietary algorithm and was categorized as unknown. The ViSi Mobile system also allows the user (patient, nurse, provider) to self-report the patient’s posture. Self-reported posture follows the same code pattern, prefixed by an “S-.” For instance, self-reported upright is “S-U90.”

The ViSi Mobile system outputs a posture recording every 15 seconds. A timestamp is used to identify the time of a given recording. A recording can be a single posture code (eg, “WLK”), or it can be a permutation of 2 or more codes (eg, “U45 U90”) if the patient performed multiple postures during the 15-second window. In the latter case, the permutation of codes is listed in the order that the postures were performed. For example, “U45 U90” indicates that the patient changed their position from reclined to upright, which is an outcome beyond the original 15 categories. Therefore, the permutation of 2 or more codes can generate many other possible outcomes of a patient’s posture recording. In the dataset, there were 185 different outcomes in the posture recordings.

A patient’s ViSi posture data are saved in several files. All files for a given patient can be identified by the patient’s medical record number (MRN). Each file usually contains data from several ViSi devices because each device needs to be charged daily and replaced with another one for continuous observation [11,12]. Within each file, each posture recording is labeled by its timestamp and the device serial number. The files vary in length, including the number of posture recordings and the number of ViSi devices.

### Data Cleaning

The study team conducted a thorough check of the data, which led to the identification of 2 problems. First, “overlap” of posture data was noted for some patients. As described previously, a given patient’s records are contained in several files identified by their MRN. For some patients, some data recordings contained in one file overlap with recordings contained in another file. These overlapping recordings have the same timestamps but different readings. They usually come from ViSi devices with different serial numbers. We call this the “overlap problem.” About 10% of patients in the dataset exhibited this problem. The overlap period can be long, up to days. Since a patient cannot wear multiple ViSi devices at the same time, overlap should not occur. We speculate that overlap could happen when a recharged ViSi device is reassigned to another patient without updating the MRN. Therefore, when overlap occurs, some data from another patient has likely been misassigned to the given patient.

Second, we identified an inconsistency between the time of hospital stay and the period of ViSi measurements for some patients. For each patient, we compared the ViSi posture data to the admission and discharge times to confirm that the patient’s data fell within this time window. However, some patients had ViSi data before their admit time or after their discharge time, which will be referred to as the “inconsistency problem.” The mismatch could be large in some cases, up to days. We believe that there are 3 possible reasons for mismatch. One possibility is that the admit and discharge times are not exact. Another is that ViSi data are sometimes collected before admission, resulting in data before the admit time. A third possibility is that the data may have come from another patient. The first 2 situations are benign, while the third situation is problematic and needs to be addressed. Based on the available information, it is not possible to determine which situation has occurred.

**Figure 1.**
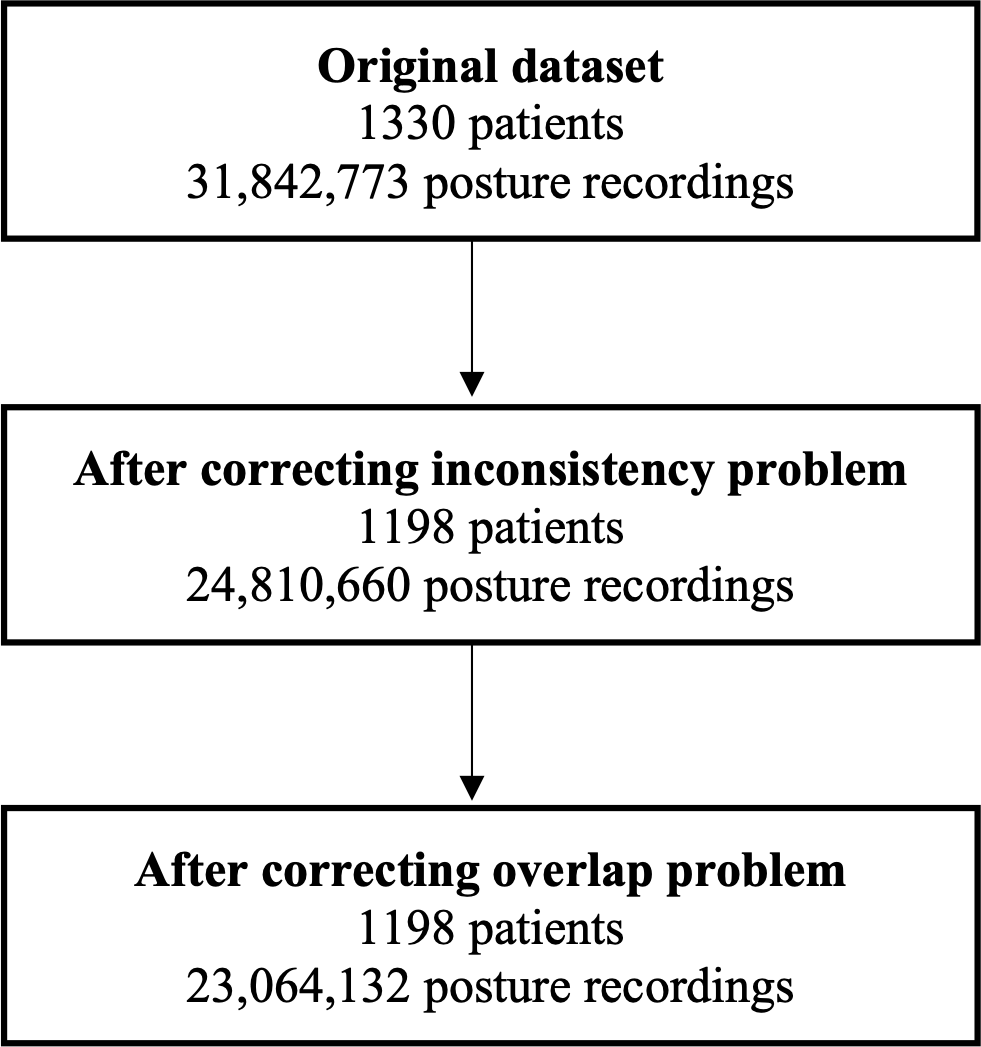
Flow diagram of data cleaning procedure. Data cleaning included two stages: (1) corrections for the inconsistency problem, and (2) corrections for the overlap problem. We show the number of patients and number of posture recordings remaining after each stage.

The following strategies were used to address these 2 problems. For the overlap problem, all overlapping records were flagged in all files for each individual patient. Data were not immediately discarded, given that one of the data streams could actually be correct. However, following discussion with in-hospital data managers and the device provider, there was no objective method to identify which data stream was correct. Therefore, this study excludes all records flagged as overlapping. A similar approach was used to address the inconsistency problem. Any data before the admission time or after the discharge time was flagged. The statistical analysis in this paper does not include flagged data. Figure 1 shows the sample size before and after we removed the flagged records for the inconsistency and overlap problems. Overall, the 2 procedures excluded 9.9% (132/1330) of patients and 27.6% (8,778,641/31,842,773) of posture recordings.

### Data Preprocessing

Before data analysis, 2 steps of preprocessing were performed to consolidate the possible posture outcomes and to remove short inconsequential fluctuations. First, we consolidated the 185 possible posture outcomes into a more manageable set of 5 groups. These five groups are Upright, Reclined, Lying, User-defined, and Unknown. Compared to the original 185 outcomes, we believe these 5 groups are more easily interpretable and useful to clinicians and analysts. In this consolidated system, each original posture recording (single code or permutation of codes) falls into 1 of the 5 groups. Table 1 provides the definitions of the posture groups, including their connections with the original posture recordings. Essentially, any posture recording that includes a self-reported code goes to the group User-defined. Any recording that includes the UNK code is classified into group Unknown. The group Lying contains posture recordings that are composed of lying codes only (ie, codes beginning with L). Of the remaining recordings, any that has a “WLK” or “U90” is put in the group Upright. The rest of the recordings must contain a “U45” and fall into the group Reclined. The third column of Table 1 shows examples of posture recordings in each group.

**Table 1.** The 5 posture groups.^a^

Posture group	Definition	Example recordings in posture group
User-defined	Any posture recording that includes “S - …”	“UNK LSP S-LSP,” “UNK LSP S-U45,” “UNK S-LLS LSP,” “UNK U90 S-U90,” “UNK U90 U45 LLS S-U90”
Unknown	Any posture recording that includes “UNK” (no “S - …”). Posture recordings that were blank were also classified as Unknown.	“UNK,” “UNK LSP”
Upright	Any posture recording including “U90” or “WLK” (no “S - …” or “UNK”)	“U90 U45,” “U90 U45 WLK,” “U90 U45 LLS,” “U90 U45 LLS WLK,” “U45 LSP WLK”
Reclined	Any posture recording including “U45” (no “S - …,” “UNK,” “U90,” or “WLK”)	“U45,” “U45 LLS,” “U45 LPR,” “U45 LPR LRS,” “U45 LRS,” “U45 LRS LLS,” “U45 LSP”
Lying	“LLS,” “LRS,” “LPR,” “LSP,” or permutations of these	“LLS,” “LPR,” “LPR LLS,” “LRS,” “LRS LLS,” “LSP,” “LSP LRS”

a LLS: lying left side; LPR: lying prone; LRS: lying right side; LSP: lying supine; UNK: unknown; WLK: walking.

Note that the Upright group combines walking with sitting and standing, rather than treating walking as a separate group. This choice was made because the ViSi device may classify the activity of walking as U90 (the same ViSi code for sitting and standing) instead of WLK, the code for walking [3]. In addition, we set User-defined as its own group, rather than assigning individual user-defined postures to their corresponding categories (eg, “S-LSP” was not assigned to Lying). This was because it is difficult to determine whether the user-defined postures were inputted intentionally. In the rest of the paper, we focus on these 5 posture groups, instead of the 185 posture outcomes. Hereafter, the term “posture” indicates posture group.

After converting the raw posture recordings to posture groups, data smoothing was performed to reduce noise. This was the second step of data preprocessing. Here, noise is defined as an isolated posture of 1 timestamp in between 2 timestamps with the same posture. For example, for a sequence of 3 postures of Lying, Reclined, Lying, the middle one is considered as noise. We removed noise through data smoothing. For each timestamp, we used the following majority vote process to remove the noise. A majority vote was taken, considering the timestamp in question and the 2 adjacent timestamps (ie, 15 seconds before and 15 seconds after). Based on the 3 votes, whichever posture appeared most frequently would be the winner. For example, if there were 2 votes for Lying and 1 vote for Reclined, the posture at the timestamp in question was taken to be Lying. If there was no winner in the majority vote (eg, the three timestamps all had different postures), then the posture at the timestamp in question was left unchanged. This process is analogous to taking a moving average using a sliding window with 3 consecutive timestamps [15], except the mode is taken instead of the average.

**Figure 2.**
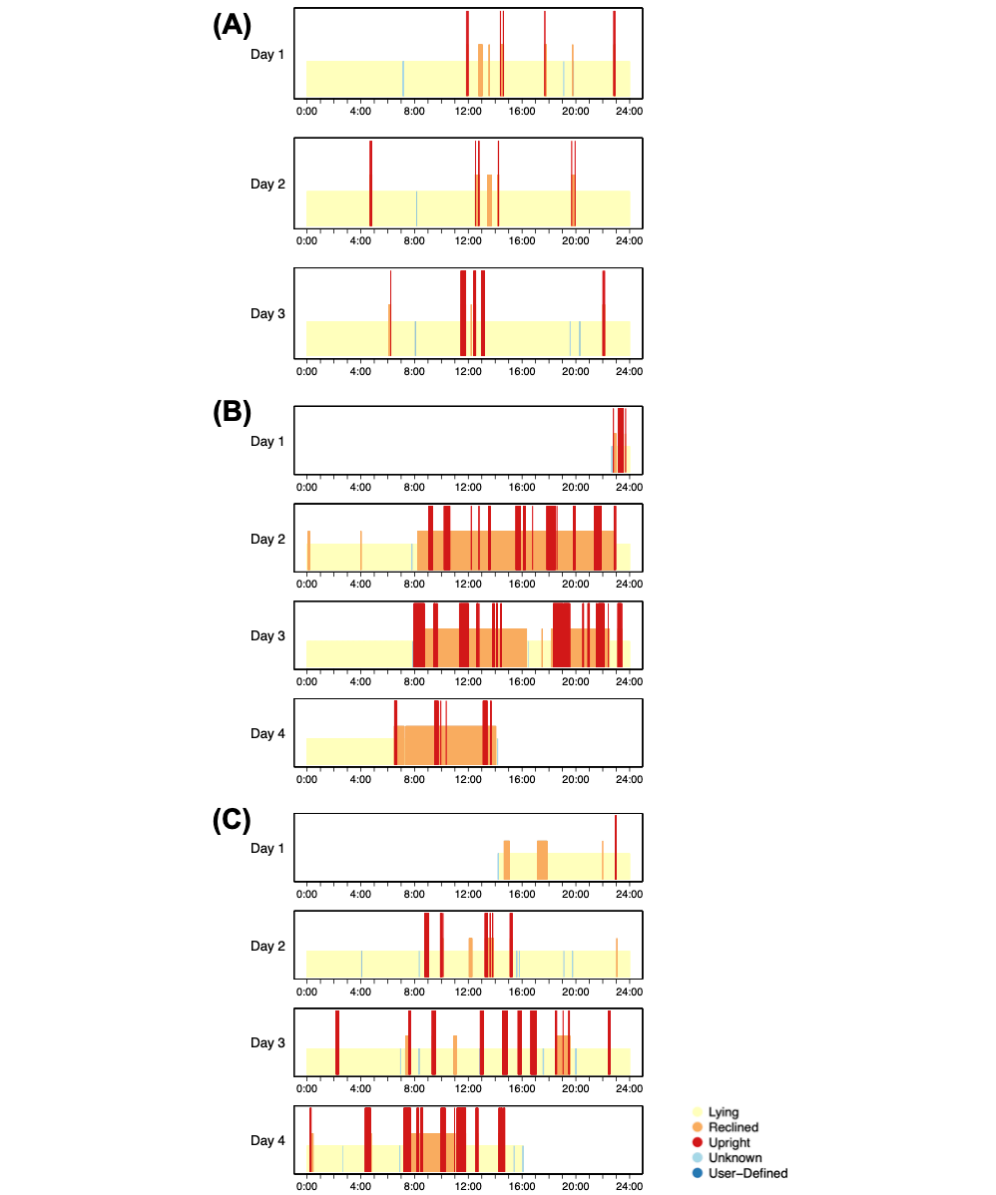
Example cleaned and preprocessed data from 3 patients. The short yellow bars correspond to Lying. The taller orange bars correspond to Reclined. The tallest red bars correspond to Upright. The light and dark blue bars correspond to Unknown and User-Defined, respectively.

Figure 2 presents 3 examples of cleaned and preprocessed posture data, which illustrate how the patterns of posture can vary from 1 patient to another. The first example (Figure 2A) shows long periods of Lying, interspersed with short Reclined and Upright segments. This patient is considered inactive. The second example (Figure 2B) shows a patient who was in the Upright and Reclined postures during the daytime hours and the Lying posture during the nighttime hours. One may infer that this patient was trying to maintain day/night routine behavior during the hospital stay. Figure 2C plots data from a cancer patient postsurgery. The patient spent days 1 and 2 mostly in the Lying posture. Increased activity was observed from day 2 to 3, with more frequent Upright and Reclined periods. On day 4, the patient stayed in Upright and Reclined postures for longer periods than previous days. This shows the patient’s process of postsurgery recovery.

The analyses described in the rest of the Methods section used the cleaned and pre-processed data.

### Summary Statistics

In all, 20 summary statistics were calculated for each patient, using their cleaned and preprocessed data. The purpose was to translate the large amount of data into a concise set of interpretable statistics. The 20 summary statistics can be divided into 3 categories. The first category (data quantity) examines the length of ViSi posture data available for the patient. The second (posture duration) finds the patient’s average time spent in each of the 5 postures. The third category (posture transitions) measures the frequency of posture changes in the patient’s data.

For data quantity, 2 measures were generated. First, we recorded the total number of days that the patient had any ViSi posture data taken (total days). Total days includes days with complete data (ie, all 24 hours), as well as days with only a short data segment starting from a single recording. Second, we counted the number of days where the patient had extensive ViSi posture monitoring, defined as at least 22 hours of recordings per day, which we used for further analysis. Hereafter, we refer to these days as analysis days. Since ViSi provides 1 posture recording every 15 seconds, each analysis day contains at least 5280 recordings. The statistics for posture duration and posture transitions, which are described below, were calculated using analysis days only. Thus, any patients who did not have any analysis days have been excluded from this analysis. Based on this criterion, 508 of 1198 patients (42.4%) were excluded. There are 690 patients included in this analysis.

For each patient, the posture duration statistics were defined as the average time spent in each of the 5 posture groups for the whole day (WD), daytime (DT), and nighttime (NT). Thus, there are 15 posture duration statistics from the 5 posture groups for WD, DT, and NT. The unit of measurement for the average time is hours per day. DT is defined as the 12-hour window from 7:00 AM-6:59 PM and NT as the opposite 12-hour window from 7:00 PM-6:59 AM. These definitions match the shift schedule at Atrium Health Wake Forest Baptist. The naming convention for the posture duration statistics is summarized in the first row of Table 2. The WD statistics provide the average number of hours per day that the patient spent in each posture. We calculated the WD statistics using the following procedure. Let k denote the number of analysis days for the patient (k≥1). Consider a posture group (eg, Lying). On each analysis day, we found the proportion of ViSi recordings that fell into the given posture. The average proportion across the k days was calculated, and the resulting value was multiplied by 24 to convert it to the unit of hours per day. This procedure was applied separately to each of the 5 posture groups, yielding a set of 5 WD statistics. Next, we calculated 2 more sets of statistics, one for DT and the other for NT. The purpose was to examine diurnal changes of the patient’s posture pattern. To calculate the DT and NT statistics, we applied the same procedure described above to the data collected during the specific 12-hour time block, except changing the conversion factor from 24 to 12. In future work, DT and NT statistics, along with WD statistics, could be used in prediction models for patient outcomes.

**Table 2.** List of statistics for posture duration and posture transitions.

	Whole day (WD)	Daytime (DT)	Nighttime (NT)
Posture duration	Lying-WD Reclined-WD Upright-WD Unknown-WD UserDefined-WD	Lying-DT Reclined-DT Upright-DT Unknown-DT UserDefined-DT	Lying-NT Reclined-NT Upright-NT Unknown-NT UserDefined-NT
Posture transitions	FPT-WD^a^	FPT-DT	FPT-NT

a FPT: frequency of posture transitions.

The third group, posture transitions, provides the frequency of posture transitions (FPT). We define that a posture transition occurs when a patient’s posture group changes from one timestamp to the next. The change is also limited among Lying, Reclined, and Upright only. Based on this definition, there are 6 types of transitions: Lying to Reclined, Lying to Upright, Reclined to Upright, and vice versa. Neither Unknown nor User-defined are considered in posture transitions. This is because whether a physical transition occurs in this case is not clear. The FPT measures the rate of posture transitions including all 6 types described above, with the unit of transitions per hour (tph). As before, we calculated FPT for WD, DT, and NT separately. The naming convention of the statistics is shown in the bottom row of Table 2. The calculation of the FPT-WD statistic is as follows. For each analysis day of the patient, we tallied the number of transitions present and divided this count by the actual number of hours of data in that day. This provided a rate of tph for this specific analysis day. The FPT-WD is the average tph across all k analysis days. The calculation of FPT-DT (or FPT-NT) uses the same procedure, except that the number of transitions and their corresponding hours are from the DT (or NT) window. Overall, 3 statistics are obtained on FPT.

### Cluster Analyses

We sought to identify the distinct subtypes of posture habits present in the dataset. For this purpose, a cluster analysis was conducted in which the patients were divided into nonoverlapping clusters, using the posture duration and posture transition statistics. These clusters can be used to identify the different subtypes of posture habits objectively. The cluster analysis was performed using the k-means clustering algorithm [16,17]. The number of clusters, k, must be specified a priori. We tested a range of values for k from 2 to 10 and selected the value of k that maximized the average silhouette width [18,19]. In total, 4 sets of cluster analyses were performed, with different input data. The first set, which we call WD cluster, used the WD statistics (Table 2, column 2). The second set (DT cluster) used the DT statistics (Table 2, column 3). The third set (NT cluster) used the NT statistics (Table 2, column 4). The fourth set (combined cluster) used the combined DT and NT statistics, which doubles the inputted data sample. In the 4 sets, we excluded posture duration statistics corresponding to the User-defined category because patients’ values for these statistics were all approximately 0, so they were not useful for differentiating between patients. Before applying the k-means algorithm, each statistic was standardized to have mean 0 and SD 1. For each of the 4 cluster analyses, average silhouette scores were computed across all patients and separately within each of the k clusters.

Analyses were performed using the R programming language (version 4.2.2; R Foundation for Statistical Computing).

## Results

### Summary Statistics

Table 3 shows the age and sex distributions of the original 1330 patients before data exclusion, and the 690 patients after data exclusion. The age and sex distributions showed little change before versus after data exclusion (Table 3). The number of patients included in the analysis was 690, comprising 407 males and 283 females, with an average age of 60 years.

**Table 3.** Age and sex distributions before and after data exclusion.

	Before data exclusion (n=1330)	After data exclusion (n=690)
**Age**^**a**^ **(years)**		
Mean (SD)	60.5 (15.5)	60.3 (15.0)
Median (IQR)	62.5 (52.0-71.8)	62.4 (52.0-71.4)
**Sex**		
Male, n (%)	772 (58)	407 (59)
Female, n (%)	557 (41.9)	283 (41)
Unknown, n (%)	1 (0.1)	0 (0)

a Age is the subject’s age on January 1, 2019.

**Figure 3.**
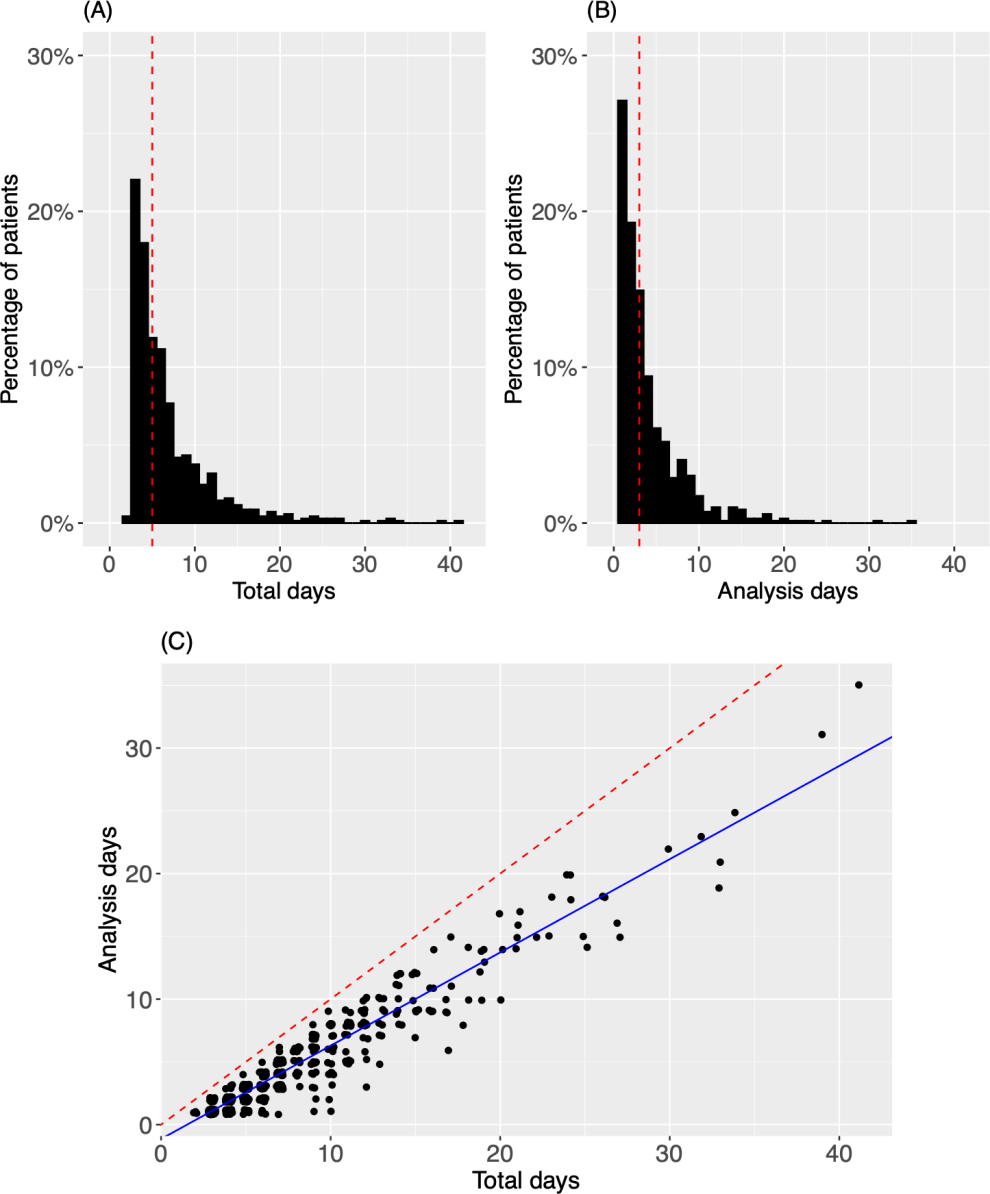
Data quantity. (**A**) The histogram for total days per subject. (**B**) The histogram for analysis days. In both (A) and (B), the dotted red line is the median of the distribution. (**C**) A scatter plot of total days versus analysis days. A linear regression fit is overlaid on the points (solid blue). For comparison, the identity line (y=x) is provided (dotted red), which represents the ideal scenario that analysis days=total days. To allow visibility of overlapping points, random noise from a uniform distribution has been added to each point in the horizontal and vertical directions.

The univariate distribution of each summary statistic was analyzed for the sample of 690 patients. Figure 3 shows the histograms of total days and analysis days, the summary statistics for data quantity. Both measures exhibit right-skewed distributions, with a higher range for total days than analysis days. The median (red dashed line) was 5 total days per subject and 3 analysis days per subject. The 2 measures are highly correlated with one another (Figure 3C). The scatter plot shows a linear relationship between total days and analysis days, estimated as:


AnalysisDays=−1.14+0.74TotalDays

As described in the Methods section, only analysis days were considered when calculating the other summary statistics reported hereafter. Approximately 85% (586/690) of patients had between 1 and 7 analysis days, including 27% (187/690) with 1 analysis day, 34% (236/690) with 2‐3, and 24% (163/690) with 4‐7 analysis days. The other 15% (104/690) had more than 7 analysis days. The maximum number of analysis days was 35.

**Figure 4.**
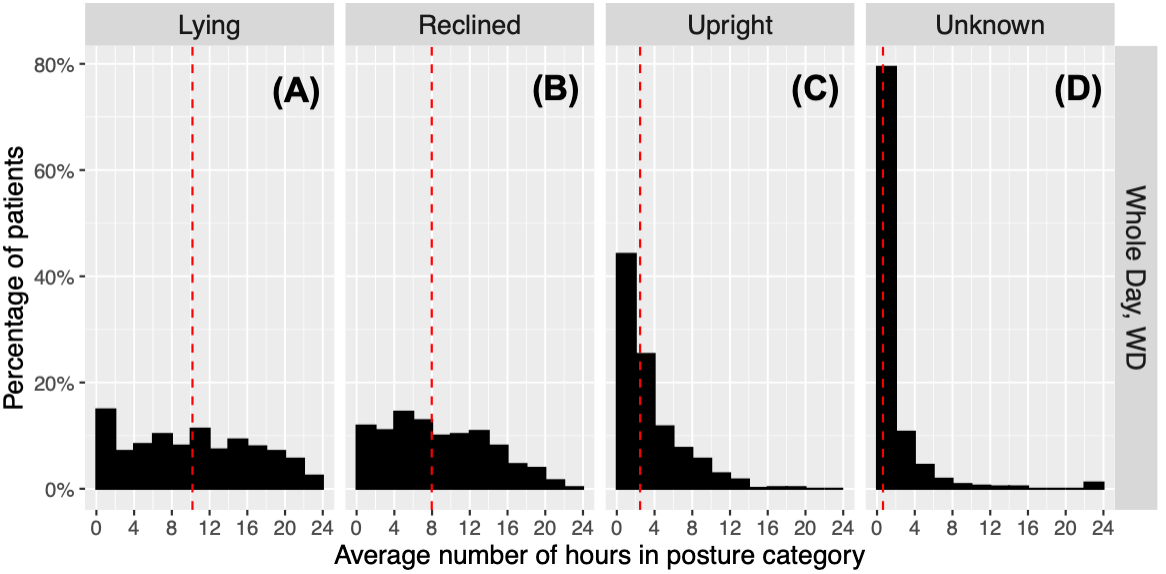
Posture duration for whole day. The histograms of the posture duration statistics are shown for whole day. The columns indicate the posture: (A) Lying, (B) Reclined, (C) Upright, and (D) Unknown. User-defined is not shown because this category was rarely observed. In each histogram, the x-axis ranges from 0 to 24 hours, and the y-axis shows the percentage of patients. The red dotted line indicates the median.

Figure 4 presents histograms of the WD statistics for posture duration, including Lying, Upright, Reclined, and Unknown. Table 4 provides the quartiles of the posture duration statistics. The histogram of User-defined is not presented in Figure 4 since it is concentrated as an isolated peak at near 0 hours/day, consistent with the median of 0.008 hours/day (Table 4). This indicates a very small fraction of User-defined posture among all patients. The distribution of Lying-WD (Figure 4A) is approximately uniform from 2-20 hours near the 9% level, with a decline after 20 hours to 2.5%. There is also a peak from 0-2 hours at 15%. This shows a wide range of time duration spent in Lying across patients. The median for Lying-WD is 10.2 hours/day. Reclined-WD shows a roughly uniform distribution from 0-14 hours at around 12%. The distribution then tapers off on the right side (Figure 4B). The median is 8.0 hours/day (Table 4). In contrast, the distribution of Upright-WD (Figure 4C) is highly right skewed, with a peak at 0‐2 hours/day accounting for about 45% (305/690) of patients. The interval from 2‐4 hours/day includes 25% (175/690) of patients. Thus, 70% (480/690) of patients spent between 0‐4 hours/day Upright. The median is 2.5 hours/day. Unknown-WD (Figure 4D) also shows a right-skewed distribution but with a sharp peak at 0‐2 hours, accounting for about 80% (548/690) of patients. The median is 0.6 hours/day. This suggests that only a small fraction of patients were characterized in the Unknown category.

**Table 4.** Quartiles for posture duration and posture transition statistics.

	Posture duration					Frequency of posture transitions (transitions/hour)
	Lying (hours/day)	Reclined (hours/day)	Upright (hours/day)	Unknown (hours/day)	User-defined (hours/day)	
Whole day, median (IQR)	10.2 (4.5‐15.6)	8.0 (4.1‐12.7)	2.5 (1.1‐4.7)	0.6 (0.2‐1.6)	0.008 (0.007‐0.009)	2.8 (1.9‐4.3)
Daytime, median (IQR)	3.3 (1.1‐6.4)	4.5 (2.4‐6.7)	1.7 (0.8‐3.4)	0.3 (0.1‐0.9)	0.004 (0.004‐0.005)	3.5 (2.3‐5.3)
Nighttime, median (IQR)	6.6 (3.1‐9.6)	3.3 (1.1‐6.3)	0.6 (0.3‐1.3)	0.2 (0.04‐0.6)	0.004 (0.003‐0.004)	2.1 (1.3‐3.5)

**Figure 5.**
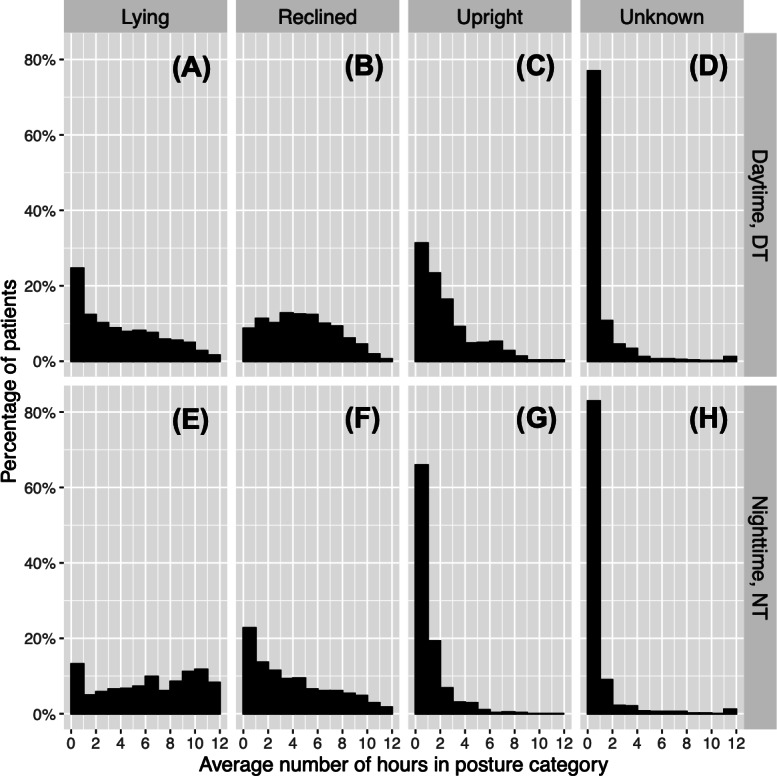
Posture duration for daytime and nighttime. This figure presents histograms of the posture duration statistics for (A-D) DT and (E-H) NT. The rows give the time of day as DT or NT. The columns indicate the posture (Lying, Reclined, Upright, Unknown). For any given row and column pair, the histogram shows the distribution of the average number of hours in the given posture during the given time of day, across the 690 patients. The x-axis of each histogram ranges from 0-12 hours. DT: daytime; NT: nighttime.

Figure 5 presents histograms of the DT and NT statistics for posture duration. Table 4 shows the quartiles of these statistics. For the Lying posture, both the DT and NT distributions were substantially different from the pattern of the WD distribution, signifying the diurnal changes of this posture. The DT distribution (Figure 5A) peaked at 0 hours at the 25% level, and then decreased monotonically from 0-12 hours. In contrast, the NT histogram (Figure 5E) increased from 1-11 hours. As expected, there is a substantially higher percentage of patients with longer Lying times during NT than during DT. Consequently, the median for NT (6.6 hours) is higher than that for DT (3.3 hours), as shown in Table 4. However, there is a noticeable peak at 0‐1 hours for NT, comprising about 13% (91/690) of patients. The Reclined distributions showed a diurnal change from roughly symmetric in DT (Figure 5B) to strongly right skewed in NT (Figure 5F). Although they had different shapes in their distribution, the median of DT (4.5 hours) was only slightly higher than that of NT (3.3 hours). The diurnal change could be explained by a certain percentage of patients changing their posture from Reclined in DT to Lying in NT, but there remained some patients who continued in Reclined from DT to NT. For Upright, the distributions for DT and NT, like that of WD, were right skewed with a peak starting at the left end. The distribution for DT has its peak of 31% at 0‐1 hours, followed by a slow decrease (Figure 5C). In comparison, the distribution of NT started from a higher peak of 66% at 0‐1 hours, followed by a sharp decrease (Figure 5G). A noticeable proportion of people spent a substantial amount of time Upright in DT. For example, 18% (123/690) of patients spent between 4‐8 hours Upright in DT, compared to 5% (32/690) in NT. The medians for DT and NT were 1.7 hours and 0.6 hours, respectively. The Unknown posture did not show a significant diurnal change.

**Figure 6.**
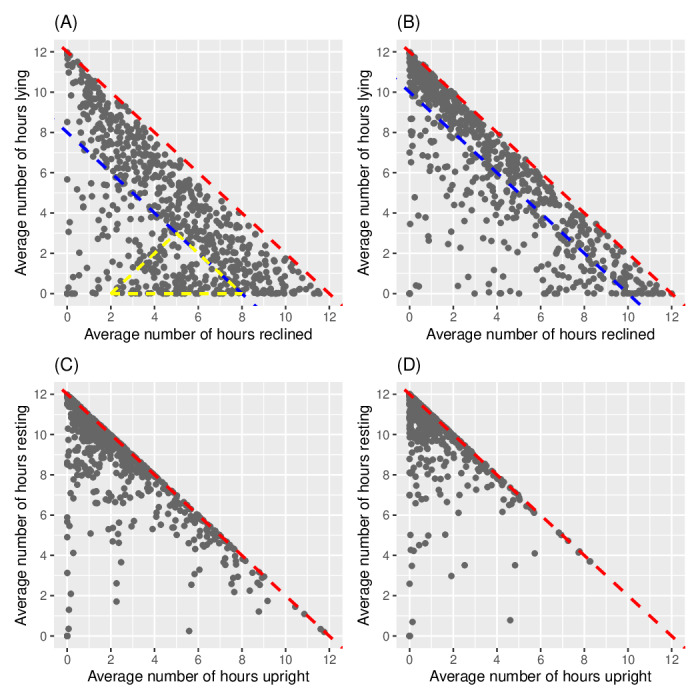
(A) Daytime: Reclined versus Lying. (B) Nighttime: Reclined versus Lying. (C) Daytime: Upright versus Rest. (D) Nighttime: Upright versus Rest. Scatter plots of time spent Lying versus Reclined (first row) and time spent Upright versus in Rest (second row). The left (right) column is for daytime (nighttime). Rest is defined as the sum of Lying and Reclined.

Interconnections between the summary statistics were also studied. Figure 6A presents a scatter plot of Reclined-DT versus Lying-DT. All points are contained inside the lower triangle of the square plot because the length of daytime is 12 hours and thus Lying-DT + Reclined-DT ≤ 12. The red dotted line (Lying-DT + Reclined-DT = 12) is the upper boundary of the triangle. Inside the triangle, points are spread out across the domain, but with a concentration at the strip between the upper boundary and lower boundary (blue dotted line) at Lying-DT + Reclined-DT = 8. This strip represents the subgroup that spends at least 8 hours Lying or Reclined during daytime. Another subarea of concentrated samples is the yellow triangle with vertices at (5, 3), (2, 0), and (8, 0). This represents a subgroup that mainly takes the Reclined posture for rest. Their total resting time is less than those in the strip. They are likely the group with more activities in daytime. Last, the subgroup in the lower left corner is sparser, with only isolated points scattered around. Figure 6B gives the scatter plot of Reclined-NT versus Lying-NT, also with a red dotted line at the upper boundary. It shows a marked change in the relationship between these two postures from daytime to nighttime. Specifically, the strip near the upper boundary of the triangle described in daytime (Figure 6A) narrows during nighttime (Figure 6B), with the lower boundary moving upward to 10 hours (blue dotted line). Most of the study population was concentrated in this strip, and the triangle mentioned previously disappeared. This is consistent with the general behavior that most people rest during the night.

For a more simplified view, Reclined and Lying can be combined into a single category called Rest, defined as the sum of these 2 postures. Figure 6C and D show the relationship between Rest and Upright in DT and NT, respectively. Most of the points are near the upper boundary (red dotted line) because patients spend most of the time in either Rest or Upright postures. In both plots, points are concentrated in the upper left corner of the triangle. The majority are in an Upright posture between 0‐4 hours and at Rest between 8‐12 hours. A diurnal change occurs since there is a concentration of points near the upper boundary with Upright between 4‐8 hours in DT (Figure 6C), while this largely disappears at NT (Figure 6D). In both plots, a small subgroup is well below the upper boundary. These points correspond to patients with higher levels of Unknown, previously shown in the right tail of Figure 5D and H.

**Figure 7.**
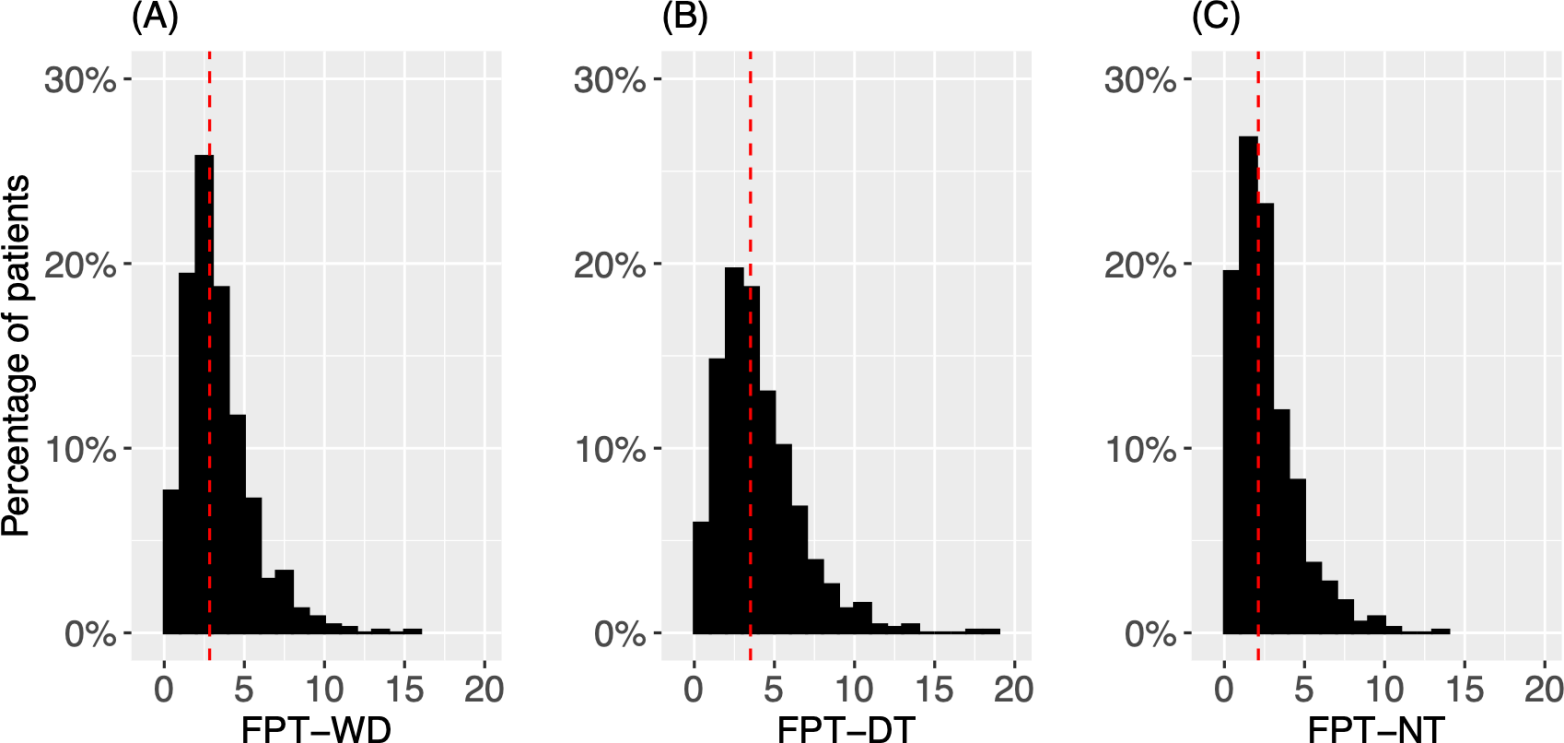
Posture transitions. (A) Histogram for FPT-WD. (B) Histogram for FPT-DT. (C) Histogram for FPT-NT. In each plot, the dotted red line indicates the median for the corresponding statistic. The unit of measurement for the FPT variable is transitions per hour. DT: daytime; FPT: frequency of posture transitions; NT: nighttime; WD: whole day.

Posture transitions were analyzed in addition to posture duration. Figure 7 shows histograms of the FPT for WD, DT, and NT. Table 4 provides the quartiles of each FPT statistic. The distribution of FPT-WD showed a prominent peak at 2 transitions per hour, accounting for 26.7% (184/690) of patients (Figure 7A). The distribution decayed quickly on both sides but with a skew to the right, shown by the long right tail extending to 15 transitions per hour. The distributions of FPT-DT (Figure 7B) and FPT-NT (Figure 7C) were also right-skewed, but showed different characteristics, reflecting a distinct diurnal change of FPT. In comparison to DT, NT had a sharper peak near 27% shifted closer to 0. About 50% (345/690) of people had 1 or 2 transitions per hour in NT. For DT, the peak was at a lower level of 20% and it decayed slower on both sides. About 35% (233/690) of people had 1 or 2 transitions per hour in DT. Moreover, the percentage of patients with higher transition rates (5‐10 transitions per hour) decreased substantially from DT to NT, demonstrated by the higher weight in the range from 5 to 10 transitions per hour in DT. Both factors contributed to the higher median for FPT-DT compared to FPT-NT (Table 4).

### Cluster Analyses

Each of the four cluster analyses divided the 690 patients into a small number of clusters. Using the criterion of average silhouette width, the number of clusters k was selected to be 5 for DT, and 4 for WD, NT, and combined. Table 5 gives the cluster averages and number of patients per cluster for WD, DT, and NT. Multimedia Appendix 2 provides the average silhouette scores across all patients and within each cluster, individually for each cluster analysis.

**Table 5.** Cluster analyses using the k-means clustering algorithm for WD cluster, DT cluster, and NT cluster. The first k rows for each cluster analysis show the k clusters in order from the largest cluster to the smallest cluster. The last column indicates the number and percentage of patients assigned to the given cluster. The other columns provide the cluster averages for the summary statistics. The last row for each cluster analysis shows the average values across all 690 patients to provide a reference point for comparing the cluster-specific means.

	Lying (hours)	Reclined (hours)	Upright (hours)	Unknown (hours)	Frequency of posture transitions (transitions/hour)	Participants (n=690), n (%)
**WD cluster**						
W1	16.7	4.3	1.7	1.3	2.5	287 (42)
W2	5.3	14.7	2.9	1.1	2.8	219 (32)
W3	6.3	9.0	7.6	1.1	5.7	161 (23)
W4	3.5	2.9	1.6	16.0	1.3	23 (3)
Average	10.2	8.6	3.5	1.7	3.3	—^a^
**DT cluster**						
D1	2.2	7.4	1.8	0.6	3.5	241 (35)
D2	7.8	2.4	1.0	0.7	3.0	226 (33)
D3	2.8	5.0	3.6	0.5	8.3	99 (14)
D4	1.0	3.8	6.7	0.6	4.1	95 (14)
D5	1.7	1.5	0.9	7.9	1.1	29 (4)
Average	3.9	4.7	2.4	0.9	4.0	—
**NT cluster**						
N1	9.2	1.8	0.5	0.4	1.9	358 (52)
N2	2.6	8.0	0.9	0.5	2.3	190 (28)
N3	3.9	4.6	3.1	0.4	5.4	116 (17)
N4	2.2	1.2	0.6	8.0	1.2	26 (4)
Average	6.3	4.0	1.1	0.7	2.6	—

a Not applicable.

In the WD cluster (Table 5), the two largest clusters (W1 and W2) spend most of the day in Rest and have low Upright time. W1 prefers the Lying posture for Rest, while W2 prefers Reclined. In both clusters, the average FPT is between 2‐3 transitions per hour. On the other hand, W3 is the most active, with the highest average Upright time (7.6 hours) and posture transition frequency (5.7 transitions per hour) among the clusters. W4 is the smallest cluster, comprising 3% (23/690) of the patients. Unknown is the dominant category in W4, with 16 hours/day Unknown on average.

The DT cluster (Table 5) identified 5 clusters using the DT statistics. Clusters D1 and D2 spend the daytime hours in Rest. D1 takes Rest mostly through Reclined at 7.4 hours on average, while D2 takes Rest mostly through Lying at 7.8 hours on average. D1 and D2 both have low levels of Upright, at 1.8 and 1.0 hours on average, respectively. D1 and D2 are close in size, accounting for 35% (241/690) and 33% (226/690) of patients, respectively. In comparison, D3 and D4 are more active during DT. D3 has an average Upright time of 3.6 hours, and it prefers Reclined (5.0 hours) over Lying (2.8 hours). D4 has a higher average Upright time (6.7 hours). It also spends more time in Reclined (3.8 hours) than Lying (1.0 hours). D3 and D4 each account for 14% of patients (99/690 and 95/690, respectively). D5 has Unknown as its dominant category and includes 4% (29/690) of patients.

The NT cluster (Table 5) found 4 clusters using the NT statistics. N1 and N2 are the two largest clusters, accounting for 52% (358/690) and 28% (190/690) of the patients, respectively. N1 has Lying as the dominant nighttime posture at 9.2 hours on average, followed by Reclined (1.8 hours). N2 prefers Reclined (8.0 hours) over Lying (2.6 hours) at nighttime. N3 has an average Upright time of 3.1 hours, which is higher than the other clusters. It also has a higher average FPT of 5.4 transitions per hour. N3 accounts for 17% (116/690) of patients. N4 has mostly Unknown posture of 8.0 hours on average.

In the combined cluster (Table 6), the traits of the clusters resemble those from the WD cluster. C1 prefers Lying, C2 favors Reclined, C3 is active in daytime, and C4 is dominated by Unknown. The sizes of C1, C2, C3, and C4 are also similar to the sizes of W1, W2, W3, and W4, respectively. Unlike the WD cluster, the combined cluster analysis revealed further details about changes in the patients’ behaviors from daytime to nighttime. For example, cluster C1 spends most of the daytime in Lying (7.0 hours), followed by Reclined (2.9 hours) then Upright (1.3 hours). At nighttime, Lying becomes more dominant (9.6 hours), with less time spent in Reclined (1.4 hours) and Upright (0.5 hours).

**Table 6.** Cluster analyses using the k-means clustering algorithm for the combined cluster.

	Lying (hours)		Reclined (hours)		Upright (hours)		Unknown (hours)		Frequency of posture transitions (transitions/hour)		Participants (n=690), n (%)
	DT^a^	NT^b^	DT	NT	DT	NT	DT	NT	DT	NT	
C1	7	9.6	2.9	1.4	1.3	0.5	0.8	0.5	3.2	1.8	291 (42)
C2	1.6	3.7	7.4	7	2.4	0.8	0.6	0.5	3.6	2.2	235 (34)
C3	1.9	4.2	4.2	4.6	5.2	2.8	0.6	0.4	6.8	5.1	141 (20)
C4	1.4	2.1	1.8	1.1	1.1	0.5	7.7	8.3	1.4	1.2	23 (3)
Average	3.9	6.3	4.7	4	2.4	1.1	0.9	0.7	4	2.6	—^c^

a DT: daytime.

b NT: nighttime.

c Not applicable.

## Discussion

### Principal Results

This study examined a year-long, routine hospital ViSi posture dataset to study patterns in patient posture habits, with the following results. The dataset required substantial preprocessing before statistical analysis. Two errors in the dataset were identified: overlap and inconsistency. Overlap was highlighted because it mixes data from multiple patients, which can alter the derived summary statistics. This problem could be easily overlooked in analyses of massive amounts of data. Overlap may have resulted from shared use of a ViSi monitoring device between multiple patients and lack of reliable timestamps of device handoff. In data preprocessing, we also consolidated the ViSi posture codes to target the most meaningful postures of Lying, Reclined, and Upright, and removed high frequency noise from the posture recordings. The preprocessing treatments improved the data quality, manageability of the data, and interpretation of results.

Using the preprocessed dataset, summary statistics were generated for each patient and probability distributions of the summary statistics were constructed using all 690 patients. This analysis showed that the 3 main postures (Lying, Reclined, Upright) had distinctive distribution patterns. For the whole day statistics, Lying had a flatter distribution, while Reclined showed a decrease in the longer hours. Upright had its peak at 0‐2 hours, with a quick monotonic decrease. Thus, Lying and Reclined are more dominant postures. These findings are consistent with known clinical observations of inpatients. Pattern changes from daytime to nighttime were observed for Lying, Reclined, and Upright. The distribution of Lying was decreasing during daytime but relatively flat with a slow increase during nighttime. Although this pattern change may be partly explained by the fact that more patients take longer Lying periods during night, the flat distribution during nighttime still needs explanation. Combined with the similarity of the daytime Lying and nighttime Reclined, we speculate that a certain number of patients take Reclined as their resting posture at night. It is also noted that over one-third of patients show a preferred duration of 3‐6 hours for Reclined posture during daytime. As expected, Upright shows a sharper decrease from daytime to nighttime.

Last, *k*-means clustering analyses were performed to objectively identify subtypes of patient posture habits. We found that 3 of the 4 analyses each identified 4 unique subtypes. These subtypes included 2 sedentary subgroups with different resting styles, one that prefers Lying and the other Reclined. There is an active subgroup that spends relatively more time Upright and has more frequent posture transitions. The fourth subtype includes a small percentage of patients that have Unknown as the dominant ViSi-measured posture during their hospital stay.

### Limitations

This study has the following limitations. First, there may still be some irreversible errors left in the data (eg, incorrect placement of ViSi device on the body, battery failure causing discontinuity), which are beyond the scope of this analysis. The postures from the ViSi device can also differ from the ground-truth postures because the ViSi postures are estimated from accelerometer data. We did our best to mitigate the issue of inaccurate posture recognition. For example, because ViSi may classify walking as U90 instead of WLK, the activities of walking, standing, and sitting were combined into the same category of Upright [3]. The difficulty in classifying walking is possibly because the ViSi algorithm, previously trained from certain data, may not fit individual patients’ mobility well. This implies that the ViSi algorithm needs to be calibrated to the individual patient, or more customized algorithms can be used to improve the accuracy of walking (eg, [20]). Second, our study does not consider postoperative pain or postoperative medications, but these two factors could affect a patient’s posture after surgery. An area of future work is to examine the effect of postoperative pain and medications on ViSi posture statistics for various types of surgery.

Third, the amount of data excluded due to overlap or inconsistency was substantial, accounting for 9.9% (132/1330) of patients and 27.6% (8,778,641/31,842,773) of posture recordings. Our study took a conservative approach of dropping suspicious data that exhibited the overlap or inconsistency problems. Although this reduced the available sample size, the number of patients included in the analysis remained high (n=690). We believe that the overlap and inconsistency problems occur randomly instead of systematically for certain types of patients. Thus, the data cleaning procedure, which removed these problems, should not cause bias. The data problems that we faced are associated with data collection, storage, and integration with the medical record. The current ViSi system is designed to provide real-time data for the nurse and clinician, rather than for long-term use. Therefore, data cleaning is an inevitable step for retrospective analyses of routinely collected ViSi data. However, some further improvements of the system could make the data more accessible for retrospective analyses. This could be done by creating standardized data models and specifications for measuring patient movement.

### Conclusions

Using ViSi posture data collected from routine inpatient monitoring, we derived summary statistics for each patient and analyzed the summary statistics to find patterns in the patient population. Probability distributions, generated from the large sample size of 690 patients, provided quantitative measures of posture durations and transitions, with respect to their median and interquartile range. We considered both surgery and nonsurgery patients in the dataset to provide a baseline measure of these quantities, which sets a basis to study more specific clinical situations. In future work, we will build on this work to study distributions for patients with specific types of disease, as well as distributions of patients before versus after surgery. After these distributions are established, they can serve as useful reference distributions to measure a future patient’s recovery trajectory or condition.

ViSi data collection increases work efficiency in the hospital. For the 690 patients analyzed in this study, the cumulative hours of ViSi posture collection amounted to 90,308 hours taken over 4847 patient-days. As an alternative, direct observation requires a nurse spending 2 hours per patient-day to conduct posture monitoring and documentation at a 30-minute frequency. Therefore, ViSi monitoring saved 9694 hours (4847 × 2) of nurses’ effort, which could be devoted to other more pressing clinical responsibilities. This corresponds to a saving of labor cost of US $401,138, based on the median hourly rate of US $41.38 per hour for registered nurses, according to the US Bureau of Labor Statistics [21]. Moreover, the digitized inpatient measurements with automated collection and storage provide a vast and valuable database for retrospective analysis, which has not been fully tapped by clinical researchers.

A major contribution of this study is development of methodology to clean and preprocess routinely collected inpatient ViSi monitoring data. The procedure we used for cleaning the ViSi posture data can have broader application in cleaning other routine hospital datasets. The preprocessing treatments for consolidating posture codes and removing high-frequency noise can also improve manageability and interpretability of ViSi posture data. Thus, this study lays groundwork for future studies on examining the associations of clinical data with posture statistics derived from cleaned, preprocessed ViSi data. In future studies, we plan to study the associations of patients’ medical condition and treatment (including diagnosis, surgical procedure, medications, sedatives, and pain level) with posture duration and posture transitions. Based on our experience with ViSi analysis, we believe that examining data quality will be the first step to ensure that clinical variables are accurately linked to the cleaned ViSi data records for each patient.

## Supplementary material

10.2196/54735 Multimedia Appendix 1 Posture codes of ViSi Mobile System.

10.2196/54735 Multimedia Appendix 2 Table of average silhouette scores for whole day, daytime, nighttime, and combined cluster analyses. For each cluster analysis, the average silhouette score across all patients is shown. In addition, the average silhouette score is provided separately for each cluster.

## Abbreviations

DT: daytime
FPT: frequency of posture transitions
LLS: lying left side
LPR: lying prone
LRS: lying right side
LSP: lying supine
MRN: medical record number
NT: nighttime
tph: transitions per hour
UNK: unknown
WD: whole day
WLK: walking
