# Supplementary material for: Using a Quality-Controlled Dataset From ViSi Mobile Monitoring for Analyzing Posture Patterns of Hospitalized Patients: Retrospective Observational Study
Source: JMIR Mhealth Uhealth. 2024 Nov 6;12:e54735. doi: 10.2196/54735 (PMC11559440; doi:10.2196/54735)
Supplement: Multimedia Appendix 1 [file mhealth-v12-e54735-s001.pdf]

Multimedia Appendix 1: Posture codes of ViSi Mobile System.

Code	Posture Category
UNK	Unknown
U90	Upright 90 degrees
U45	Upright 45 degrees
LSP	Lying supine
LPR	Lying prone
LRS	Lying on right side
LLS	Lying on left side
WLK	Walking
FALL	Fall
S-U90	User selected upright 90 degrees
S-U45	User selected upright 45 degrees
S-LSP	User selected lying supine
S-LPR	User selected lying prone
S-LRS	User selected lying on right side
S-LLS	User selected lying on left side
