# Supplementary material for: Using a Quality-Controlled Dataset From ViSi Mobile Monitoring for Analyzing Posture Patterns of Hospitalized Patients: Retrospective Observational Study
Source: JMIR Mhealth Uhealth. 2024 Nov 6;12:e54735. doi: 10.2196/54735 (PMC11559440; doi:10.2196/54735)
Supplement: Multimedia Appendix 2 [file mhealth-v12-e54735-s002.pdf]

Multimedia Appendix 2: Table of average silhouette scores for WD, DT, NT, and Combined cluster analyses. For each cluster analysis, the average silhouette score across all patients is shown. In addition, the average silhouette score is provided separately for each cluster.

**(A) WD Cluster**		
Cluster	***Average Silhouette Width***	
W1	0.47	287 (42%)
W2	0.36	219 (32%)
W3	0.16	161 (23%)
W4	0.37	23 (3%)
**All patients**	**0.36**	**690 (100%)**
**(B) DT Cluster**		
Cluster	***Average Silhouette Width***	
D1	0.35	241 (35%)
D2	0.45	226 (33%)
D3	0.16	99 (14%)
D4	0.36	95 (14%)
D5	0.39	29 (4%)
**All patients**	**0.36**	**690 (100%)**
**(C) NT Cluster**		
Cluster	***Average Silhouette Width***	
N1	0.51	358 (52%)
N2	0.41	190 (28%)
N3	0.14	116 (17%)
N4	0.41	26 (4%)
**All patients**	**0.42**	**690 (100%)**
**(D) Combined Cluster**		
Cluster	***Average Silhouette Width***	
C1	0.41	291 (42%)
C2	0.30	235 (34%)
C3	0.12	141 (20%)
C4	0.30	23 (3%)
**All patients**	**0.31**	**690 (100%)**
